# Early Development of a Virtual Coach for Healthy Coping Interventions in Type 2 Diabetes Mellitus: Validation Study

**DOI:** 10.2196/27500

**Published:** 2022-02-11

**Authors:** Giulia Bassi, Ivan Donadello, Silvia Gabrielli, Silvia Salcuni, Claudio Giuliano, Stefano Forti

**Affiliations:** 1 Department of Developmental and Socialization Psychology University of Padova Padova Italy; 2 Centre Digital Health & Wellbeing Fondazione Bruno Kessler Trento Italy; 3 KRDB Research Centre Free University of Bozen-Bolzano Bolzano Italy

**Keywords:** virtual coach, early development, type 2 diabetes mellitus, healthy coping, Wizard of Oz, ORBIT model, pilot study, mobile phone

## Abstract

**Background:**

Mobile health solutions aimed at monitoring tasks among people with diabetes mellitus (DM) have been broadly applied. However, virtual coaches (VCs), embedded or not in mobile health, are considered valuable means of improving patients’ health-related quality of life and ensuring adherence to self-care recommendations in diabetes management. Despite the growing need for effective, healthy coping digital interventions to support patients’ self-care and self-management, the design of psychological digital interventions that are acceptable, usable, and engaging for the target users still represents the main challenge, especially from a psychosocial perspective.

**Objective:**

This study primarily aims to test VC interventions based on psychoeducational and counseling approaches to support and promote healthy coping behaviors in adults with DM. As a preliminary study, university students have participated in it and have played the standardized patients’ (SPs) role with the aim of improving the quality of the intervention protocol in terms of user acceptability, experience, and engagement. The accuracy of users’ role-playing is further analyzed.

**Methods:**

This preliminary study is based on the Obesity-Related Behavioral Intervention Trial model, with a specific focus on its early phases. The healthy coping intervention protocol was initially designed together with a team of psychologists following the main guidelines and recommendations for psychoeducational interventions for healthy coping in the context of DM. The protocol was refined with the support of 3 experts in the design of behavioral intervention technologies for mental health and well-being, who role-played 3 SPs’ profiles receiving the virtual coaching intervention in a Wizard of Oz setting via WhatsApp. A refined version of the healthy coping protocol was then iteratively tested with a sample of 18 university students (mean age 23.61, SD 1.975 years) in a slightly different Wizard of Oz evaluation setting. Participants provided quantitative and qualitative postintervention feedback by reporting their experiences with the VC. Clustering techniques on the logged interactions and dialogs between the VC and users were collected and analyzed to identify additional refinements for future VC development.

**Results:**

Both quantitative and qualitative analyses showed that the digital healthy coping intervention was perceived as supportive, motivating, and able to trigger self-reflection on coping strategies. Analyses of the logged dialogs showed that most of the participants accurately played the SPs’ profile assigned, confirming the validity and usefulness of this testing approach in preliminary assessments of behavioral digital interventions and protocols.

**Conclusions:**

This study outlined an original approach to the early development and iterative testing of digital healthy coping interventions for type 2 DM. Indeed, the intervention was well-accepted and proved its effectiveness in the definition and refinement of the initial protocol and of the user experience with a VC before directly involving real patients in its subsequent use and testing.

## Introduction

### Background

Technology is revolutionizing both the self-management and self-care of people with diabetes mellitus (DM) [1] by developing digital solutions for the monitoring of blood glucose levels, diet, and physical exercise [2]. However, those based on fostering mental health and emotional symptoms have mainly been developed for the general population [2]. According to the American Association of Diabetes Educators (AADE) guidelines, people with DM can feel depressed, anxious, and stressed, and these emotions can affect the management of their disease [3]. Indeed, AADE guides on 7 areas regarding the healthy management of DM, such as physical activity, monitoring of blood glucose levels, healthy eating, taking prescribed medication, reducing risk behaviors, problem-solving skills, and healthy coping [3]. Altogether, these 7 areas have been included in the National Standards for Diabetes Self-Management Education, referring to the framework of patient-centered diabetes management [4]. The latter area, namely healthy coping, is related to psychological support, in which its key factors are the achievement of healthy coping strategies and the maintenance of a positive attitude toward the management of DM [3]. Therefore, reaching healthy coping skills is fundamental as these allow them to acquire healthy goals. In this regard, diabetes-related emotional distress can influence the motivation of people with DM to self-manage their disease; when this motivation diminishes, the clinical recommendations requested for better self-care are difficult to maintain [3]. Indeed, experiencing diabetes-related emotional distress can inhibit the self-care behaviors of people with DM. For example, studies have found that quality of life shows a significantly positive association with good metabolic control and a negative relationship with diabetes complications [5,6]. Moreover, several psychosocial factors have been identified as risk factors in reducing the capacity of people with DM to maintain metabolic control because of diminished treatment adherence [7]. These psychosocial factors refer to experiencing stressful events, symptoms of depression, family stress, low financial resources, low social support, and the use of maladaptive coping strategies [8,9]. Indeed, adopting and cultivating healthy coping skills allows these people to be more adherent to clinical recommendations, as well as in other areas of life, such as following a healthy diet and engaging in regular physical activity [3].

### Virtual Coaches: Related Studies

Virtual coaches (VCs) are generally computer programs that simulate conversations with people by mimicking a human being [2]. More specifically, VCs have mainly been deployed for coaching behavioral strategies. VCs are often developed within messaging apps, websites, or mobile phone apps, and they can interact using various methods, such as SMS text messages, images, audio tracks, and video clips [2]. VCs in the field of DM are mostly focused on the monitoring of blood glucose levels, diet, and physical activity, such as Wellthy, developed within a smartphone-based app [10]. Interestingly, a recent systematic review identified only 2 VCs that were specifically designed for the monitoring of healthy management of DM. One of the VCs was developed for diabetes diagnosis [11], whereas the other VC comprised a variety of chronic diseases, including DM but specifically focused on psoriasis [12]. Monitoring tasks are the most common features of VC approaches for DM [13]. For example, the My Diabetes Coach program is an app-based embodied conversational agent named Laura developed to support diabetes self-management in a home setting over 12 months. This app focuses on several modules, such as the monitoring of blood glucose levels, healthy eating, taking medication, physical activity, and the importance of foot care. This app further includes a scale for assessing the health-related quality of life [14]; however, the app is not addressed to the improvement of this outcome, and the construct of healthy coping has not been considered in the development of Laura. Given this, it is worth noting the relevance of developing a VC to support people with DM in their healthy coping. In this regard, a VC has been established for older adults with type 2 DM (T2DM), using the voice speech of Google Home, taking into account the construct of healthy coping [15]. Individuals can interact with the VC through this voice speech, which is able to initially screen depression symptoms using the Patient Health Questionnaire-9 [16], and then respond to them with personalized suggestions regarding the monitoring and maintenance of blood glucose levels. In general, VC approaches are still unable to handle the emotional needs of individuals with DM [17] as their main goal is to deliver digital interventions without creating a personalized interaction between the VC and the user. Indeed, the only personalized feedback based on people’s data is typically related to insulin dosage suggestions [18,19]. Furthermore, people with DM have certain expectations associated with app design, such as being engaging, incorporating activities recommended by the clinical guidelines (eg, AADE), and covering a broad range of content, including psychological and emotional support [20], by taking into consideration their motivational state of change, which is useful in delivering personalized psychological interventions (Transtheoretical Model of Change); therefore, more evidence is required. Most VCs are developed for smartphone-based apps rather than social media communication channels. In this latter case, for example, the use of Telegram, WhatsApp, or SMS text messaging [21] is widely used by the general population, and, indeed, people have already integrated them into their daily lives. On the other hand, mobile health (mHealth) apps present more issues, such as high dropout rates. For example, two-thirds of people who downloaded an mHealth app used it only once [22]. Indeed, the change of habitual behavior requires a person to invest a large amount of time and thus, benefit from this technology for a sufficient period to incorporate them into their daily lives [22]. Overall, the psychological support that VC approaches offer to the user still represents important challenges for VC solutions. More specifically, the design of psychological digital interventions that are acceptable, usable, and engaging for the target user still represents the main challenge. Finally, the comprehension of emotions, feelings, tone, irony, sarcasm, and therefore the interpretation of the intended user meaning has proved to be a pivotal challenge in the development of a VC [23].

### The Design of VCs: The Wizard of Oz Methodology

The development of a robust, natural, and personalized VC is necessary to achieve a wide-ranging, conversion-specific protocol, which reflects the users’ needs and preferences in the intervention dialogs with the VC [24-28]. The typical approach is to collect primary stimuli from these resources, such as Wizard of Oz (WOZ), refine them with the users’ interactions, validate them with some experts in the field, and then repeat the procedure to reach a protocol of the intervention, which is as acceptable and as effective as possible [24-27,29,30]. Initially, the prototyped intervention should be sufficiently straightforward to ensure an easy evaluation of the user experience (UX) and its refinement to fulfill users’ needs [31]. Therefore, the WOZ methodology requires three fundamental elements: first, a script or protocol, which guides what should happen during the interaction; second, the presence of participants who play the role of the final user, thereby using the role-playing technique; and finally, a human operator, called Wizard, who will be the one to perform the virtual coaching tasks in a simulated scenario, whereas at the final stage, these tasks will be automated [32]. The WOZ method is a process that allows users to interact with a counterpart without knowing that the answers are generated by a human being (ie, also called human-controlled) rather than a computer. This deceit aims to let the users experience a greater freedom of expression [32]. A previous study that used the WOZ methodology for the design of a VC for stress detection among older adults showed how this method allows the development of a more natural dialog flow, as well as the importance of the users’ needs in designing the intervention [33]. Therefore, one can assume that a VC can offer a convenient, engaging way of providing psychological support to people anytime and anywhere.

### The Design of VCs: Standardized Patients Approach

In designing a VC, the WOZ method assumes the presence of people who apply the role-playing technique, which can be combined with the standardized patient (SP) approach. SPs are actors trained to play the role of a patient, thereby simulating a problem in a clinically relevant and realistic way [31,34]. In particular, this approach is useful when an intervention is not yet mature, and thus, testing the protocol in different scenarios of simulation can be more useful and quicker than involving real patients, who are vulnerable user groups. The intention is not to use the representation of an SP to replace an actual encounter with a real patient but to supplement it in an integrative and standardized method [34,35].

To our knowledge, this is the first study that uses the WOZ and SP approaches in the early development of a VC for psychological support among people with DM. In this regard, the digital health technologies available in the literature, whose main aim is to support people in adopting and cultivating positive coping strategies, are still in their infancy. However, the progress of technology has allowed the opportunity for the development of automated support, such as VC approaches.

### Objective

The main objective of this study is to develop VC interventions based on psychoeducational and counseling approaches to support and promote healthy coping behaviors in people with T2DM.

In particular, in the early development of VC, the specific objectives are as follows:

1. To develop an initial healthy coping intervention protocol for psychological digital interventions delivered by a VC to people with T2DM
2. To validate the conversational protocol with mental health and behavioral intervention technologies (BITs) experts
3. To test the prototype of VC and thus the intervention protocol with participants who play the role of an individual with T2DM through the use of the WOZ methodology
4. To adapt and refine the VC based on the results that emerge in this study regarding acceptability, usability, and UX
5. To assess user engagement and the accuracy of the users’ role-playing by means of clustering techniques applied to the logged conversations between WOZ and participants

The overall data will be useful in future work to convert the WOZ into a conversational agent developed with an automatic VC implemented on neural networks. The VC will be prepared for a proof-of-concept study with patients with DM [36] by making the dialogs as natural and effective as possible to future target users.

### Participant Recruitment

Participants in the *Define* phase were selected for the development of the intervention protocol. The only criterion for eligibility was the presence of a team of psychologists.

Participants in the *Refine* phase were 3 experts in mental health and BITs.

Each team works at the Digital Health Lab, the Fondazione Bruno Kessler (Italy), and the Department of Developmental Psychology and Socialization of the University of Padova (Italy).

Participants in the *Refine* phase were 20 psychology students recruited from the University of Padova (Italy). Of the 20 students, 2 (10%) dropped out from the study; thus, the final sample comprised 18 (90%) psychology students (14/20, 78% women), with ages ranging from 19 to 28 (mean age 23.61, SD 1.975) years, who played each 1 out of the 6 SP roles assigned. The participants were randomly recruited by telematics means; that is, by email to the university students’ mailing list. The inclusion criteria for the study were (1) being a student in psychology, as they were expected to be better able to empathize with the characteristics, feelings, and needs of the SP profiles assigned and (2) being familiar with smartphones and the WhatsApp messaging channel.

## Methods

### Study Design

#### Overview

**Figure 1:**
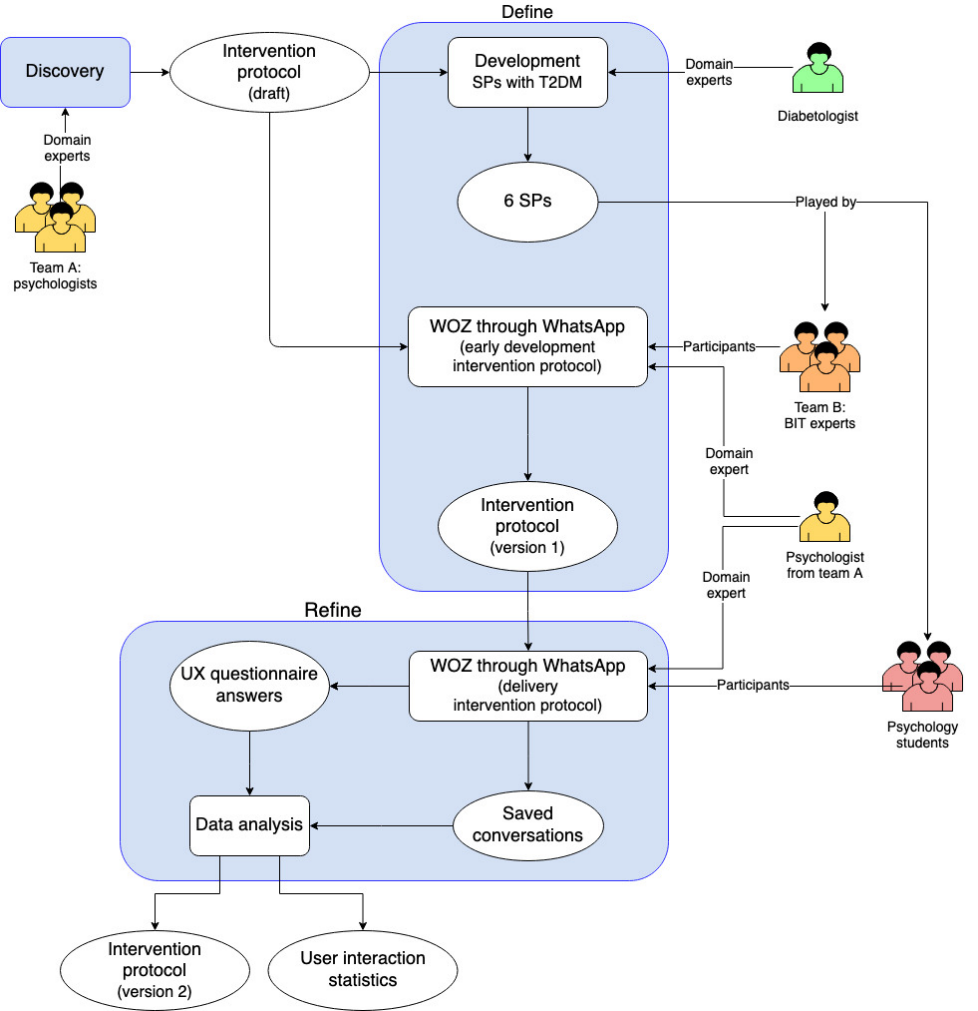
The study design architecture developed in this study. Rounded rectangles represent the architecture components or performed activities, whereas the ellipsis represents the input and output of each activity. SP: standardized patient; T2DM: type 2 diabetes mellitus; WOZ: Wizard of Oz; BIT: behavioral intervention technology; UX: user experience.

The design method of this study was 2-fold, and the architecture of the study design is shown in Figure 1.

First, we used the WOZ method, in which the users thought they were interacting with a VC but were interacting with a human being. This deceit allows the participants to express a language that is as natural as possible.

Second, we used the Obesity-Related Behavioral Interventions Trials (ORBIT) model as a guide for the development of this study. The ORBIT model has been developed for behavioral treatment to prevent or manage chronic diseases, such as DM [37,38]. Indeed, for this study, ORBIT allowed for guiding the development of healthy coping interventions by setting clear objectives for each phase of the model, thus enabling the revision of previous phases to improve the intervention in line with the new findings [37]. As an early development and validation study, we followed the first 3 phases of the ORBIT model, which are described in the following sections.

#### Phase 1: Discovery

##### Overview

As seen in Figure 1, the first phase of our study design was the development of the first draft of the intervention protocol. This process involved background research in behavioral and cognitive psychology, health psychology, the cornerstones of motivational interviewing, and the field of human-computer interaction, with a specific focus on the health care domain. A recent review reported that, in the overall studies, psychological support is underresourced and sometimes inadequate, thereby leading to poor health-related quality of life and decreasing the general well-being of people with DM [39]. Therefore, the main goal of this study was to develop an intervention protocol as part of the implementation of a VC (named Motibot, the abbreviation for Motivational bot) in a messaging system for psychological support to overcome the associated barriers of nonadherence in the context of DM. In this regard, a team of psychologists (team A) developed an intervention protocol based on counseling and psychoeducational approaches focusing on the healthy coping construct. This protocol comprised 2 sessions per week for an overall length of 6 weeks. At the beginning of each session, the VC delivers motivational and behavioral support to improve individuals’ motivation to change their behaviors by adopting healthy coping strategies to handle the struggle caused by diabetes-related psychological symptoms. More specifically, motivational interventions are based on dialogs aimed at increasing individuals’ awareness of their emotions and on the costs and benefits of adopting a healthy behavior, favoring their psychophysical well-being. Behavioral support is based on video clips or audio tracks inspired by positive psychology interventions and mindfulness practices. Mindfulness exercises are related to mindfulness-based cognitive therapy [40]. In this scenario, the support of VC to the user is not intended as a therapy but rather as psychoeducational support to reach, adopt, and cultivate healthy coping strategies for better diabetes management.

##### Phase 1a: Define

The *Define* phase represents the next step in our study design architecture. Here, the early development of the intervention protocol was performed by involving 3 mental health and BITs experts (team B), who knew who the WOZ was (from team A). These experts interacted with the WOZ by playing the role of 50% (3/6) developed SP profiles using the WhatsApp messaging channel. These 6 profiles were created in collaboration with a diabetologist of the Padova Hospital (Italy), and they represented the profiles of 3 (50%) typical male and 3 (50%) typical female patients with T2DM, which was suitable for every age (ie, youths, adults, and older adults) as they are the target users of the VC. Examples of SPs are shown in the Multimedia Appendix 1. The experts were consulted to define the intervention protocol regarding (1) the psychological approaches targeting DM, (2) the psychoeducational intervention, and (3) the UX assessment tools; indeed, an open-ended question was included to capture additional thematic content related to the UX. The team of experts defined and refined the intervention protocol following an iterative process, including recent scientific evidence based on cognitive behavioral interventions. Therefore, the intervention protocol (version 1) was defined for the next phase.

##### Phase 1b: Refine

In this phase, as a preliminary test, the WOZ delivered the intervention protocol to psychology students through the WhatsApp messaging channel. The intervention protocol was implemented for 6 weeks, with 2 sessions per week. This phase represents the initial pilot testing of the previously designed protocol. First, participants were asked to interpret and simulate an SP with T2DM by using the role-playing technique during the testing phase. Notably, 6 SP profiles were randomly assigned to the participants. Before starting the intervention, participants were told that they would interact with a VC. In fact, they interacted with a human being called WOZ (from team A). At the end of the second, fourth, and sixth weeks, users filled in the UX questionnaire, in which the items were adapted from the original UX questionnaire [40]. Qualitative data were further collected from the users’ answers to the open-ended question, “I’m asking you to express your opinions regarding your experience with me...” At the end of the present phase, the intervention protocol (version 2) was refined based on the users’ answers regarding their experience with the VC.

**Figure 2:**
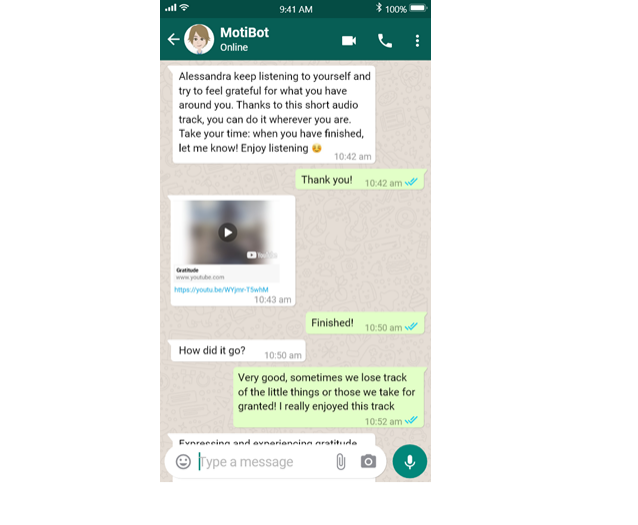
Example of the interaction between WOZ and a user within the WhatsApp application.

Finally, all the conversations between WOZ and users were saved, and subsequently, the data analysis stage allowed for processing of the intermediate results that emerged in the study (ie, to assess the user interaction statistics). This work will allow the training of a conversational agent to play the role of the simulated VC. An example of the interaction between the WOZ and a user is shown in Figure 2.

### Measures

To accomplish the purposes of this study, we administered the following tools described in the following paragraphs.

*The UXQuestionnaire* was developed from the original version of the UX Questionnaire [41] to make the bipolar adjectives in line with the goals of this study. In particular, it includes 26 adjectives, either positive or negative, to assess the experience of interacting with the VC. Each item was rated on a 5-point Likert scale ranging from 1 (strongly disagree) to 5 (strongly agree). At the end of the questionnaire, an open-ended qualitative question was added and formulated as follows: “I’m asking you to express your opinions regarding your experience with me...” This latter question allows users to freely express their thoughts about their experiences with the VC. Textbox 1 shows the selection of items for this study.

The Mood Rating Scale was administered to support them in being aware of their emotions using the following question: “What are you feeling at this precise moment?” They were further asked to indicate the intensity of their emotions on a 5-point Likert scale, from 1 (low) to 5 (high).

Data collection and the relative timing during the 6-week intervention are reported in Textbox 2.

**Positive and negative items of the user experience questionnaire.**

**Positive items**

- Pleasant
- Profound
- Cordial
- Comprehensible language
- Empathetic
- Attentive
- Motivating
- Encouraging
- Supportive
- Trustworthy
- Flexible
- Interesting

**Negative items**

- Annoying
- Not reliable
- Unappealing
- Unclear
- Complicated
- Not efficient
- Too much information
- Dissuading; not stimulating
- Not engaging
- Unpredictable
- Not reflective
- Conventional
- Rigid

**Tools administered and their timing.**

**User experience questionnaire**

- At the end of the second, fourth, and sixth week

**Mood Rating Scale**

- At the beginning of each dialog session (2 per week) between the Wizard of Oz and the participants

### Procedure

Over the 6 weeks of the study, participants were free to interact at any time with the WOZ, who was proactive in starting each session (typically at 10 AM). The UX questionnaire was developed in Google Forms, which was sent via a link through WhatsApp. The study procedure was conducted in compliance with the Declaration of Helsinki (Italian law 196/2003, European Union General Data Protection Regulation 679/2016) and was approved by the ethical committee (3518; April 1, 2020) for psychological research at the University of Padova (Italy). All participants signed and returned the informed consent document before participation and after revealing the WOZ identity at the end of the study.

### Data Analyses

Statistical analyses were performed using R (version 64; R Foundation for Statistical Computing) [42], SPSS Statistics (version 24.0; IBM Corp) [43], and Python (version 3.6.6; Python Software Foundation) [44].

We used a mixed methods approach, which combines quantitative and qualitative analyses.

The Shapiro-Wilk test was performed to assess the normal distribution of the answers to the items related to the UX questionnaire.

Bartlett test was further conducted to evaluate homoscedasticity among the participants. The assumption of homoscedasticity, which means the same variance, is the main aspect of linear regression models [45]. Homoscedasticity explains a situation in which the error term (defined as the noise or random interference between the independent variables and the dependent variable) is equal across the overall values of the independent variables [46].

The first 2 objectives of this study (ie, the development of an intervention protocol for healthy coping in DM and its validation with mental health and BITs experts) were performed during the *Discovery* and *Define* phases, respectively. The third objective (ie, intervention protocol testing using the WOZ method among university students), which refers to the *Refine* phase, allowed us to further achieve a fourth objective: the evaluation of user acceptability, usability, and UX. Therefore, the statistical analyses performed to reach this goal were formulated as given in the following paragraphs.

The main *descriptive statistics* (ie, means and SDs) were conducted regarding the following:

1. Descriptive statistics were used for the number of utterances and their length (ie, the number of characters) expressed by both the VC (ie, WOZ) and users. Each utterance is a user’s sentence sent via WhatsApp. These utterances are provided as a text file by WhatsApp with the
  Save conversation
  function. Each line of this file contains an utterance, its time stamp, and the name of the relative user. These descriptive statistics are necessary to highlight the differences between the VC and user messages to further understand whether the VC is too informative or too demanding from users. The users’ response time (evaluated in minutes) was also assessed to understand the acceptability of the VC intervention.
2. The item response distributions among the 26 items of the UX questionnaire were performed to understand the users’ experience with the VC every fortnight (ie, second, fourth, and sixth weeks).

*A series of 1-way repeated measuresanalysis of variance* was conducted to explore whether there were significant differences in each item’s means of the UX questionnaire every fortnight (ie, second, fourth, and sixth weeks). The significance level α=.05 was Bonferroni corrected to α=.0025.

*Qualitative analyses* were conducted by referring to the analysis of the user’s immediate feedback on their experience with the VC, collected at the end of the UX questionnaire. Qualitative contents were analyzed using the thematic analysis method developed by Braun and Clarke [47], thereby following their 6 phases. The approach is inductive and experiential in its orientations and is essential in its theoretical framework. First, the authors read and reread the users’ answers to become familiar with the data and thus identify potential themes. Second, 2 authors (ie, GB and SS) analyzed and coded the thematic content, identifying labels for each theme relevant to the aim of this study. Third, the analysis conducted by the aforementioned authors allowed the searching of themes regarding the users’ quotes, which was consistent with the scope of this study. Fourth and fifth, the authors (GB and SS) proceeded with the quality checking of themes related to the coded data and the entire data set. Finally, they reviewed the qualitative responses to be included in the final report.

On the other hand, the statistical analyses performed to reach the fifth and, thus, the last objective, namely the assessment of users’ engagement and the accuracy of the users’ role-playing, were formulated as given in the following paragraphs.

The *Augmented Dickey-Fuller* test was used to check whether the length (ie, the number of characters) of the users’ answers or utterances and their response times presented a trend according to the dialog steps. If the trend was not detected, users were reasonably engaged in conversations with the VC. A decreasing trend in the length of the users’ answers or an increasing trend in response times can be interpreted as a signal of disengagement.

Clustering techniques (ie, unsupervised learning based on the K-means algorithm) were used to assess the accuracy of the users’ role-playing. Here, K-means is applied to vectors of real numbers gathered from the embedding of user utterances. Sentence embeddings are natural language processing techniques that can map natural language sentences or utterances into vectors of real numbers to preserve their semantic distance. Therefore, if 2 user utterances have a similar meaning, the Euclidean distance of the corresponding embedding vectors will be very low. The embeddings are computed with the Italian version of an off-the-shelf trained neural network called the universal sentence encoder [46]. This neural network is state of the art in sentence embedding. The results are based on the Fowlkes-Mallows Index (FMI), in which higher values indicate greater similarity between the computed clusters and the true assignment users—SPs. A great similarity means that users’ utterances are in line with the given SP; that is, they correctly performed the role-playing. Therefore, the harvested dialogs can be automatically clustered according to their role, and consequently, they represent a good starting point for training a VC based on neural networks.

## Results

Overall, the users’ answers showed a nonnormal distribution. There were no missing data, and every participant answered all the questions presented in the UX questionnaire.

### Descriptive Analyses

#### VC and Users: Utterances and Response Time

Table 1 shows the number of VC and user utterances along with the number of characters per utterance and the users’ response time for each of the 18 dialogs.

With regard to the number of utterances, the VC expresses twice as many utterances compared with that of the users, as shown by the means and 50%. In addition, the length of the VC messages (ie, number of characters per utterance) was more than double that of the users. This is aligned with the original intention of co-designing a virtual coaching intervention for healthy coping. In particular, these findings are in line with the role of the VC, which is proactive and guides the user in self-reflecting upon their emotions to learn how to adopt healthy coping strategies in dealing with negative thoughts and feelings.

Regarding the users’ response time, half of the users answered on average within 15 minutes, whereas some users only answered within 2.33 minutes and others within 81 minutes. These results indicate a good acceptance of the VC intervention and commitment in the study, which is also confirmed by the low number of dropouts from the study (ie, 2/20, 10% users).

**Table 1:** Descriptive analyses of the utterances and response times within the dialogs.

Measure	Number of VC^a^ utterances	Number of users’ utterances	Number of VC characters per utterance	Number of users’ characters per utterance	Users’ response time (minutes)
Values, mean (SD)	244.39 (19.05)	122.5 (17.7)	86.23 (3.14)	27.98 (9.7)	27.57 (27.20)
Values, minimum	219	95	79.91	13.06	2.23
25th percentile	231.25	111.5	84.4	18.9	5.24
50th percentile	240	120.5	85.96	30.31	15.45
75th percentile	257.25	130	87.74	35.45	48.28
Values, maximum	283	174	93.61	41.55	81.55

^a^VC: virtual coach.

#### UX Questionnaire: Positive Item Response Distributions

**Figure 3:**
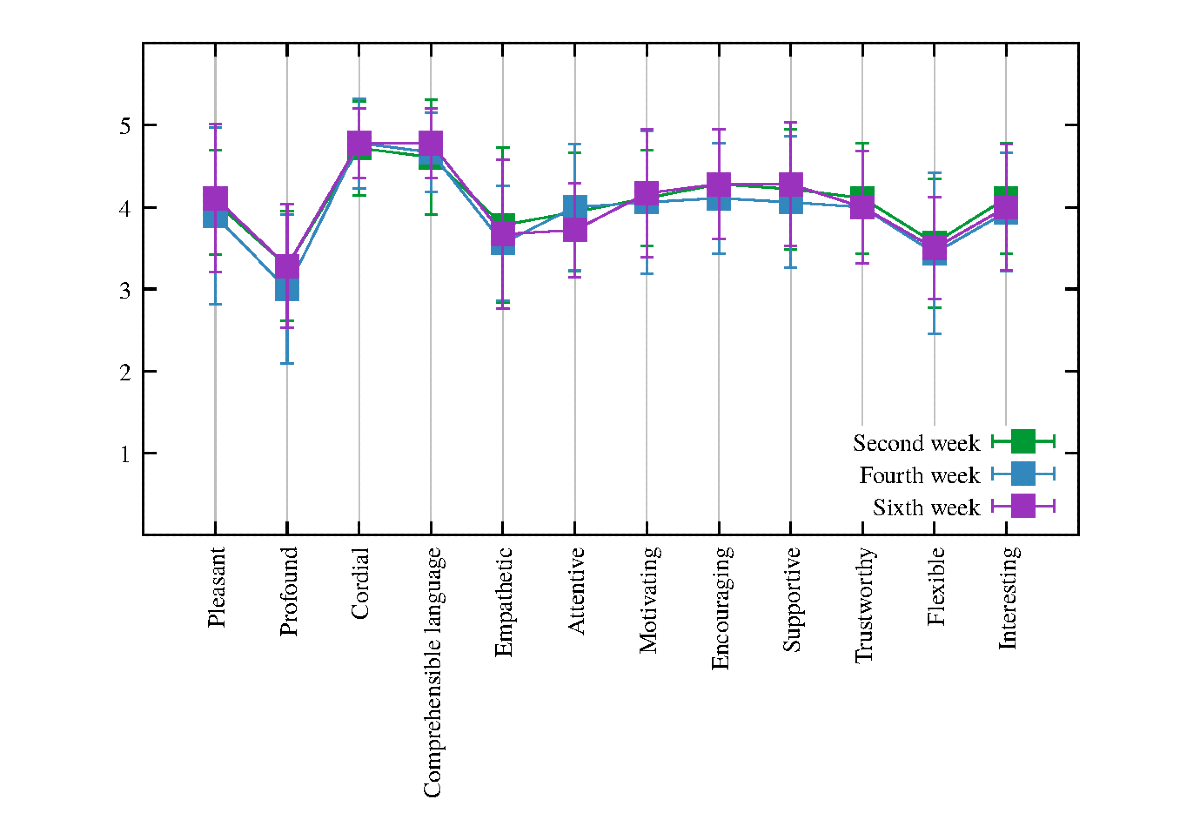
Plot of the positive items every fortnight week (second, fourth, and sixth week). Square dots and error bars correspond to means and SDs, respectively (N=18).

As displayed in Figure 3, overall, the positive items from the UX assessment showed a mean >3 on a 5-point Likert scale (mean 4.02, SD 0.72). In particular, the items *comprehensible language*, *motivating*, *encouraging*, and *supportive* slightly increased every fortnight week (second, fourth, and sixth week). The precise means and SDs are reported in Multimedia Appendix 2 (Table S1).

#### UX Questionnaire: Negative Item Response Distributions

**Figure 4:**
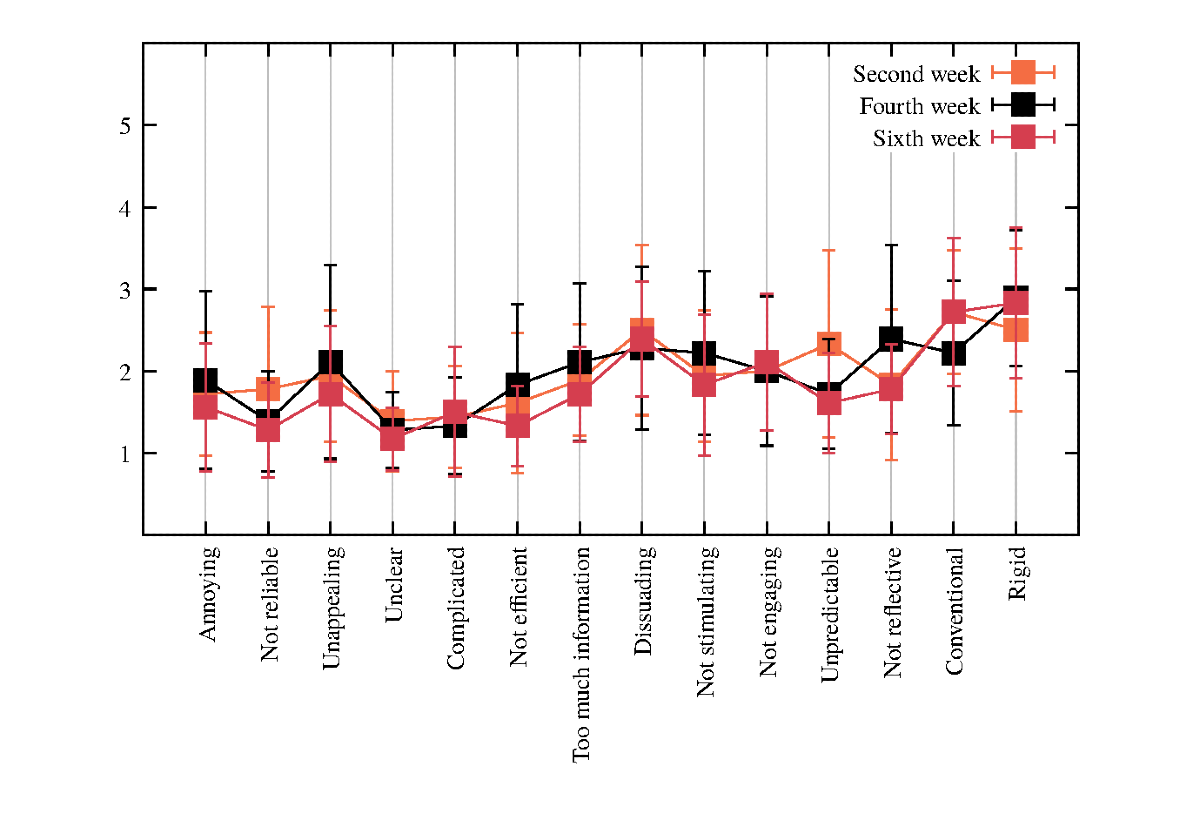
Plot of the negative items every fortnight week (second, fourth, and sixth week). Square dots and error bars correspond to means and SDs, respectively (N=18).

Overall, the mean of the negative items of the UX questionnaire was in the expected direction (mean 1.92, SD 0.81), whereby all the items decreased with the progress of the intervention, as shown in Figure 4. Of particular note is the item *not reflective*, which decreases in week 6 compared with weeks 2 and 4. Moreover, the item *unpredictable* had a decreasing trend over the fortnight weeks, whereas the item *conventional* increased. Finally, the item *rigid* slightly increased in week 4 and slightly decreased in week 6. Again, the precise means and SDs are reported in Multimedia Appendix 2 (Table S2).

### Analysis of Variance Repeated Measure: Differences in the UX Answers

A series of 1-way repeated measure analysis of variance with Bonferroni corrections were performed to compare items of the UX questionnaire on the second, fourth, and sixth week. The results showed no significant differences between the variables. The only significant differences were found for 2 negative items, *not efficient* (*F*_1,652_=3.752; *P*=.03) and *too much information* (*F*_1,906_=3.974; *P*=.03), in which their means decreased during the fortnight week. These differences were detected as significant by post hoc tests.

### UX From a Qualitative Perspective

Table 2 shows the qualitative data gathered from participants’ free text answers to the UX questionnaire, sent at the end of the second, fourth, and sixth weeks. This process allowed us to better understand their interaction with the VC and thus their UX. Two main categories were identified by two judges (GB and SS), resulting in (1) *insight themes* comprising the subcategories of *pleasant*, *natural*, *supportive*, *stimulating*, and *self-reflective* and (2) *challenge themes* comprising the subcategories of *repetitiveness* and *restrictive*.

**Table 2:** Users’ quotes to the open-ended question provided at the end of the user experience questionnaire for each week (second, fourth, and sixth week).

Weeks and users’ quotes		Insight themes	Challenge themes	
**Week 2**				—^a^
	“I think it is a pleasant and useful reminder to think about your mood and emotional aspects.” [Participant 3]	Self-reflective		
	“I find the experience stimulating.” [Participant 4]	Stimulating		
	“I find this new approach very interesting because without being intrusive, it enables you to have the right support during the day and proposes stimulating activities.” [Participant 7]	Supportive		
	“The messages stimulate a lot of self-reflection in the present moment. The responses to the messages seem very much in line with what I wrote.” [Participant 14]	Natural		
**Week 4**				
	“I am enjoying the interaction. It is not too forced and often the exchange is pleasant.” [Participant 7]	Pleasant	—	
	“I found it to make very stimulating proposals and always try to stimulate a person even when they are not so inclined to undertake a certain activity.” [Participant 9]	Stimulating	—	
	“Interesting but sometimes a little repetitive in the advice and suggestions.” [Participant 10]	—	Repetitiveness	
	“It seems to respond adequately to my answers. When I answer more articulately its answer is often in line with what I wrote.” [Participant 14]	Natural	—	
	“I think this is a good way to support. If you follow the suggestions consistently, I think it can be very helpful.” [Participant 15]	Supportive	—	
**Week 6**				
	“Motibot offers many interesting and not trivial ideas on how to cultivate your well-being. I think it can be very useful, especially for those who do not know this area. Moreover, beyond the content of the messages, I think that having a regular appointment during which you have to stop for 10/15 minutes and think, can have many positive implications. The only criticism, which comes to my mind, and which is perhaps intrinsically linked to the origin of Motibot, is that its ‘sensitivity’ to the answers of the writer could be improved because sometimes one gets the impression that it has not ‘understood.’ Despite this, however, since it is a virtual entity, I believe that it performs its function correctly.” [Participant 1]	—	Restrictive	
	“This path with Motibot has been very positive and useful to take an optimistic, comprehensive, and effective perspective; besides listening to me more, managing my emotions better and taking care of myself as well as others. As a result, I understood how to achieve greater well-being, paying attention to the present moment, and getting in tune with the world.” [Participant 6]	Self-reflective	—	
	“I found the proposed activities very interesting and useful to practice; I think it is good support as it can motivate and encourage. Maybe sometimes I found some proposals and related explanations a bit repetitive among them, but overall, the interaction was very pleasant.” [Participant 10]	—	Repetitiveness	
	“It was very stimulating.” [Participant 12]	Stimulating	—	
	“This bot can help, and support people diagnosed with diabetes. The answers are very much in line with what I wrote. If you write a long and articulate message, the bot will understand if that message is positive or negative and will respond accordingly. Only a few times, it happened that the answers seemed a little out of place. The exercises that are proposed to you are easy to understand and do not require too much time. In my opinion, this allows the person not to interrupt the path, and to follow the advice without too many problems in their working day and not.” [Participant 14]	Natural	—	
	“It was a positive experience, gradually I felt more and more involved and motivated to listen to the tracks and the proactive suggestions.” [Participant 17]	Supportive	—	

^a^Not available.

### The Augmented Dickey-Fuller Test as an Insight for User Engagement

Table 3 displays the results of the Augmented Dickey-Fuller tests for each user. A very low test statistic indicates that the average number of users’ characters of their utterances (ie, second column) or their response times (ie, fourth column) does not change significantly according to the dialog steps. Users did not tend to give shorter utterances and their response times did not increase, thereby demonstrating that the engagement of the users remained stable. These findings show that all users, except 1, engaged in interaction with the VC.

Regarding the average number of characters per user utterance, the test presents a low statistic for all users, except for user 14. This later showed a lower critical value of −3.49 at 1%; indeed, this user wrote longer sentences in the last utterances with the VC, thereby assuming a higher engagement with it.

**Figure 5:**
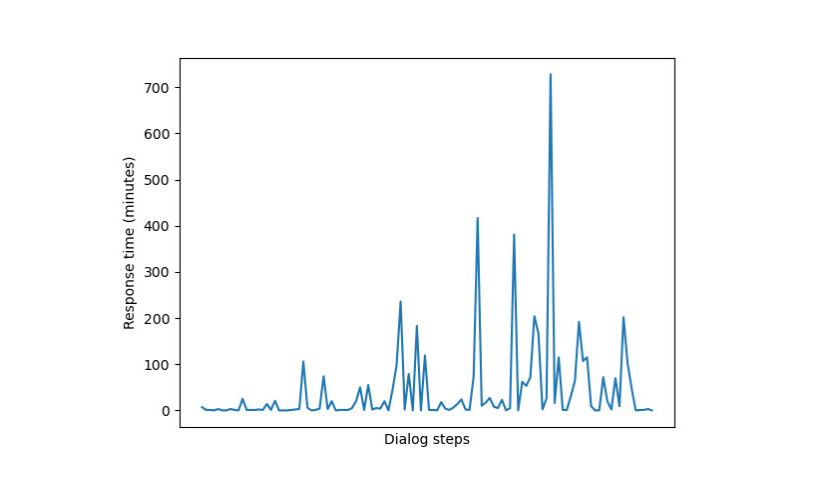
The response times for user 5 according to the dialog steps.

With regard to the average response time per user, the test has good results for all users, except for users 5, 11, and 15 (critical value of −3.5% at 1% for all). During the last interactions with the VC, users 11 and 15 received longer behavioral interventions (ie, audio tracks and video clips), which required more time to understand the content and, consequently, to react to it. Therefore, even if these cases present a slight increasing trend in response time, they cannot be properly addressed as disengagement. Indeed, in these cases, users may need more time to reflect and answer the VC. In addition, their test statistics were quite close to the critical value, and the *P* value was <.05. At the end of the study, user 5 replied with a longer time, remaining as the one who presented a longer response time. This trend can be easily verified by inspecting the plot of the time series of the response times, as shown in Figure 5. Notably, the increasing trend starts from the central part of the dialog. Therefore, all users, except 1, tended to be engaged in answering with a nonincreasing response time. The intent was not to have a quick response time but to explore whether there was a response time with a nonincreasing trend.

**Table 3:** The statistical test and corresponding *P* values for the Augmented Dickey-Fuller test regarding the average number of characters and the average response time per user.

User	Number characters, mean		Response time, mean	
	Test statistics	*P* value	Test statistics	*P* value
1	−8.63	5.96×10^–14^	−9.55	2.65×10^–16^
2	−9.42	5.57×10^–16^	−10.22	5.30×10^–18^
3	−10.12	9.68×10^–18^	−9.57	2.25×10^–16^
4	−9.44	4.89×10^–16^	−11.04	5.50×10^–20^
5	−8.97	7.85×10^–15^	−1.94	.31
6	−11.18	2.52×10^–20^	−10.8	2.01×10^–19^
7	−11.66	1.89×10^–21^	−10.77	2.40×10^–19^
8	−10.22	5.35×10^–18^	−11.63	2.23E–21
9	−10.31	3.22×10^–18^	−10.28	3.78E–18
10	−9.81	5.68×10^–17^	−12.33	6.40E–23
11	−10.6	6.26×10^–19^	−3.36	.01
12	−6.77	2.71×10^–09^	−9.49	3.59×10^–16^
13	−7.12	3.84×10^–10^	−12.1	1.99×10^–22^
14	−3.24	.02	−10.88	1.30×10^–19^
15	−5.76	5.63×10^–07^	−3.36	.01
16	−5.55	1.64×10^–06^	−10.07	1.30×10^–17^
17	−9.82	5.36×10^–17^	−10.5	1.07×10^–18^
18	−5.65	9.98×10^–07^	−9.73	9.18×10^–17^

### Clustering Techniques: Assessing the Accuracy of Users’ Role-playing

Table 4 shows the cluster results obtained using the K-means algorithm. The second column shows the true user role ID assignment, hereafter referred to as the gold standard. The third column shows the clustering run by the algorithm. The names of the SP profiles were removed from the dialogs to avoid biases in the neural network performing the embedding. According to Table 4, all users who played role A were clustered, except for user 0. The same holds for role user 4. Users who played role C were split, and user 7 was clustered with user 6, who played a different role. Users who played role E were grouped correctly. FMI is defined as higher values indicating better performance. In this study, FMI=0.32 indicated quite low performance. However, if we remove the users who played the SP of *Mirta* (Mirta is a woman aged 70 years with T2DM; see the whole description in the Multimedia Appendix 1), we thereby obtain an FMI of 0.72, which indicates a high similarity between the cluster and gold standard. These results confirm that the users who role-played Mirta’s profile did not play that profile accurately. On the other hand, the remaining users played their roles satisfactorily.

**Table 4:** The K-mean results (third column) compared with the true role assignment (second column) for each user in the first column.

User ID	Gold standard role ID (name)	Predicted role ID
0	A (Alessandra)	cluster_0
1	A (Alessandra)	cluster_1
2	A (Alessandra)	cluster_1
3	A (Alessandra)	cluster_1
4	B (Alessandra)	cluster_2
5	C (Federico)	cluster_3
6	C (Federico)	cluster_4
7	D (Federico)	cluster_4
13	E (Simona)	cluster_3
14	E (Simona)	cluster_3
15	E (Simona)	cluster_3
16	E (Simona)	cluster_3
17	E (Simona)	cluster_3

## Discussion

### Overview

To date, many studies have developed behavioral interventions among adults with T2DM, mainly concentrating on the monitoring of their diet, physical activity, and blood glucose levels. In addition, the literature has proliferated, with studies emphasizing the extent to which psychosocial symptoms such as anxiety, stress, depression, and diabetes-related emotional distress directly affect diabetes management and glycemic levels [48]. These studies suggest the importance of including these symptoms in the development of interventions among people with DM [48]. Moreover, technology has opened up the potential for new types of interventions, such as mHealth solutions or VC approaches. Bearing all these aspects in mind, there is a need for more effective and accessible technology-based interventions, such as those delivered through VCs, to improve the self-care and self-management of people with DM. Moreover, the development of a robust, natural, and personalized VC is required to support the psychological needs and coping skills of people with T2DM to ensure adherence to clinical recommendations and motivate them to achieve and adopt healthy coping strategies when they experience symptoms of stress, anxiety, and/or depression.

### Early Development, Acceptability, and UX

With regard to the first 4 aims, this study showed how an intervention protocol for healthy coping in the context of T2DM was formalized using both the recommendations of 3 mental health and BITs experts and the results of this study. This pilot study, as a preliminary test, highlighted how the ratio between the number of VC utterances and the number of user utterances provided us important feedback regarding the VC capability of allowing self-reflection. Indeed, a ratio close to one indicates an underinformative VC and, therefore, a low self-reflection role, whereas a high ratio indicates an overinformative VC, resulting in undue stress for the users.

The simulated VC presented a length of dialogs twice as long as the user’s answers, which is in line with the design of the proactive VC-driven intervention. Indeed, the VC aims to trigger users’ self-reflection on their emotions by teaching them healthy coping strategies, such as mindfulness exercises for better coping with chronic diseases. Furthermore, the VC relies on the Mood Rating Scale, in which users can reflect on and express what they feel every day and, subsequently, be aware of their emotions. On the other hand, the current VC intervention does not require too much text input and information from users, with a low mean of 27.98 (reported in Table 1) regarding the number of characters for user utterances; thus, it makes the VC less demanding but, at the same time, engaging enough for the user. In addition, the low SD in the VC utterances represents useful feedback for its improvement. A more tailored approach might involve different intervention pathways according to the users’ characteristics, such as their age, gender, level of education, and habits. With regard to the users’ response time, the advantage of the VC is that it gives users the right time to think and reflect on the proposed motivational and behavioral interventions; indeed, some users need more time than others to answer. Furthermore, the average of the positive items was above the median value of 3, and the negative items were under the median of 3. These results suggest that the perception of VC is positive, and it is going in a promising direction. Moreover, of particular interest are the items *motivating*, *encouraging*, and *supportive*, which moderately increased during the 6 weeks, thereby showing a positive and engaging interaction of users with the VC. With regard to the negative items, *not reflective* and *unpredictable* decreased in week 6 compared with weeks 2 and 4, showing that the simulated VC was perceived as thoughtful and predictable as every fortnight week progressed. The item *conventional* increased, indicating that users understood the direction of the dialogs delivered by the VC and felt more confident and secure in interacting with it. Finally, the interaction seemed to become slightly more *rigid*, which may be because of the setup of the intervention protocol. Only the items regarding *not efficient* and *too much information* showed a significant difference, in which their means highlighted a decrease, thereby demonstrating that the VC presents appropriate support to the user without being too intrusive or sending too much information. These latter results are in line with the statistical analyses conducted on the number of utterances and number of characters, which refer to the users’ dialogs with the VC.

### Users’ Quotes

Qualitative analyses well-confirm the aforementioned quantitative results of this study, showing a correspondence between the items of the UX questionnaire and the themes that emerged from the users’ responses to the open-ended question, adding other important considerations. There are several lessons learned, starting from the users’ open-ended answers to the UX questionnaire to the feedback gathered from the interaction between the VC and users.

The users’ responses to UX allowed an in-depth understanding of the users’ perception regarding the interaction with the VC. Therefore, the VC was found to be supportive, allowing a certain level of self-reflection on the participants’ own emotions, and natural and pleasant in its dialogic interaction. It was also found to be a little restrictive in its interaction and sometimes repetitive in its suggestions but good at stimulating thinking and supporting the use of new coping strategies. On the basis of these results, several refinements of the intervention protocol were conducted. With regard to motivational content, in addition to dialogs to increase motivation to adopt healthy coping strategies, we also included exercises that allow people to adopt a positive attitude toward diabetes management. An example of a simple and short exercise is thinking about a positive episode, describing it, and expressing the associated emotion. The motivational content of the third week was also changed by focusing on mindfulness to aspects related to the quality of sleep among adults with DM from a psychological perspective. Moreover, the frequently asked questions, collected from the users’ conversation, and the possible responses were also added to the intervention protocol, such as the following:

- “What did you mean with gratitude?”
- “Aren’t you going to send me a video or a recording today?”
- “How can I improve my motivation?”
- “What strategies will you teach me to be more mindful?”

### User Engagement and the Accuracy of the Role-playing Technique

With regard to the last aim, which was to evaluate the users’ engagement with the VC and the accuracy of their role-playing technique, some interesting results emerged.

All users, except 1, were actively engaged with the VC: they did not shorten their answers to the VC or increase response times as a result of a repetitive or uninteresting interaction. These results confirmed a stable and positive engagement level with the VC intervention. Moreover, the clustering algorithm demonstrated that most users played the assigned role accurately, interpreting the role effectively, even when personalizing it. This is also confirmed by the fact that they did not use the same words for the same role. The clustering analysis showed that the role-playing technique was performed accurately in most cases; indeed, high accuracy emerged in clustering users according to their roles. For instance, Cohen et al [49] found that the SP approach represents the most effective method for primary care physicians and reduces the proportion of patients with uncontrolled asthma by 27%, thereby improving their self-management. Regarding the SP approach, findings that emerged in this study showed that users found difficulties in interpreting Mirta’s role. This result may be because of age differences between the SP and users. Indeed, as previously mentioned, Mirta was a woman aged 70 years, and, on the other hand, users presented a mean age of 23.61 years. Therefore, they could have encountered some physiological difficulties in interpreting that role; indeed, none of them played the role accurately. When the dialogs collected are grouped into roles, these dialog clusters further represent an initial data set for training a VC based on neural networks, meaning that they are able to respond to users more adaptively over the intervention. To this extent, the promising results that are presented here regarding the neural networks in the clustering analysis suggest that these algorithms can discriminate between the different SP roles. Therefore, they can potentially respond to personalized interventions according to the specific SP profiles. The results of this study highlighted how the use of the WOZ and SP approaches, combined with the role-playing technique, can prove useful in the early validation of VCs for patients with chronic diseases.

### Limitations and Strengths

Pilot testing was performed among university students instead of real patients with DM; however, this choice was considered appropriate. This choice was guided by the need to improve the intervention protocol from the UX perspective without compromising their well-being. The adoption of the WOZ and SP approaches allowed us to iteratively refine our intervention before presenting it to real patients for larger testing in the field. This process allows the collection of several interesting insights from users, which is useful for developing future versions of the VC prototype. This perspective is also in line with the approach recommended by the ORBIT model (in particular for phases 1a and 1b) to implement increasingly more effective interventions with the involvement of relevant stakeholders in the design process. Finally, the use of clustering techniques to assess the accuracy of users’ role-playing represents one of the more innovative contributions of this study.

### Conclusions

The preliminary results of this study are promising, and the tested VC intervention was well-accepted. Indeed, most users found the VC useful for better coping with emotions and negative thoughts related to the burden of managing DM. Users further showed a good acceptance level for the VC intervention, especially for self-reflection on their emotions. The use of the WOZ and SP approaches allowed effective and rapid refinement of the intervention protocol. The overall results provide the opportunity to train an algorithm to replace the real coach as the training data quality has been checked, which shows that it is possible to capture the mean stream behaviors of each role. This training will refer to phase 2 of the ORBIT model, which is a proof-of-concept study. This study will include real patients with DM to evaluate the efficacy of the psychoeducational intervention, which will be delivered via the Telegram messaging channel [36,37]. The VC will assess symptoms of anxiety, stress, depression, health-related quality of life, and diabetes-related emotional distress at preintervention, postintervention, and follow-up to measure the intervention’s efficacy. UX and user engagement will be further assessed. Thus, the ambition of the VC in its new version is to leverage the relevant patients’ profiles to personalize content and dialogs delivered and achieve a more effective and higher-quality intervention.

## Appendix

Multimedia Appendix 1 Examples of standardized patient case scenarios for every age (ie, youths, adults, and older adults).

Multimedia Appendix 2 Means and SDs of the positive and negative items every fortnight week (second, fourth, and sixth week; N=18).

## Abbreviations

AADE: American Association of Diabetes Educators
BIT: behavioral intervention technology
DM: diabetes mellitus
FMI: Fowlkes-Mallows Index
mHealth: mobile health
ORBIT: Obesity-Related Behavioral Interventions Trials
SP: standardized patient
T2DM: type 2 diabetes mellitus
UX: user experience
VC: virtual coach
WOZ: Wizard of Oz
