# Supplementary material for: Early Development of a Virtual Coach for Healthy Coping Interventions in Type 2 Diabetes Mellitus: Validation Study
Source: JMIR Form Res. 2022 Feb 11;6(2):e27500. doi: 10.2196/27500 (PMC8881774; doi:10.2196/27500)
Supplement: Multimedia Appendix 1 [file formative_v6i2e27500_app1.docx]

Multimedia Appendix 1

Table S1. Examples of Standardized Patient Case Scenario for every age (ie, youths, adults, and older adults)

**Youth**

Alessandra is a sunny and creative woman of 25 years, who has just finished the *“Accademia delle Belle Arti”* in Venice. Her passion is sculpture, and she says she will be thrilled to show her work at an exhibition one day.

Alessandra is short in stature and overweight. Since she was a child, she says she has always been a bit shapely and in particular she has always loved sugars, especially chocolate.

Alessandra recounts an episode in which, having one a sculpture competition, she celebrated with her best friend by sharing a whole jar of chocolate spread and a packet of biscuits, claiming “you always celebrate on a full stomach”.

Here, the problem is that her food portions are too large in relation to her limited physical activity; Alessandra reports that she occasionally goes jogging on the embankment near her home but has never been consistent in her sport.

Alessandra reports that last year, for almost four months, she would wake up very thirsty and during the night she had difficulty sleeping because of the continuous waking up to go to the toilet.

Alessandra decided to go to her general practitioner who prescribed a serious of blood tests, which revealed that she is suffering from Type 2 Diabetes Mellitus.

Therefore, Alessandra had to change her diet, and this has caused her some difficulty; indeed, she says: “giving up sugars is not easy, especially for a sweet tooth like me! I have to find a solution, like sport, but I have never been very consistent”.

**Adult**

Simona is a woman of 40 years. She is of average height and stature. Her posture is rigid, and her facial features are slightly angular. She has been working as a secretary in a dental practice for 20 years and she claims that she would never change her job: the boss is kind and polite, the environment is sunny, and she knows almost all the patients with whom she has established good relationships.

She considers herself a reasonable person, who knows how to keep her behaviour under control. She reports that she does physical activity once a week, particularly aerobics, in the gym next to her house; indeed, she says “I enjoy it a lot and I also care about my health, as the Latins say *mens sana in corpore sano!*”.

Six months ago, one of her monthly medical check-ups—since she is very health conscious—she discovered she had Type 2 Diabetes Mellitus. She reports that she has a family history of diabetes: her grandmother suffered from Type 2 Diabetes Mellitus, which later led to her blindness.

Simona reports that she did not need to make many lifestyle changes, but still feels that she has to be even more careful about her health and follow her doctor’s recommendations.

Therefore, Simona decides to take care of herself by continuing to eat healthily and to exercise regularly, but she feels that something has changed. This feeling brings her a certain discomfort, which she defines as fear, she says “I am afraid of the complications that diabetes can cause and the same thing that happened to my grandmother can happen to me; this thought is quite present, it really stresses me out! How do I stop it?”.

**Older Adult**

“Mirta is an elegant and good-looking woman of 70 years. Mirta is of average height but slightly overweight, which is also due to her age that makes it difficult for her to regularly exercise.

Mirta lives alone, however she has two daughters who visit her every weekend in order to take her for a stroll, although they often have to convince her to go out. In the last four months Mirta reported that she feels a bit lazy and prefers to spend her afternoons reading a good novel while sitting in an armchair. Her daughters have noticed a certain lack of interest in taking care of herself: this behavior would have seemed unlikely a short time ago.

Mirta discovered that she suffers from Type 2 Diabetes Mellitus at the age of 52. However, her lifestyle has not substantially changed since then, except for her concern about frequently checking her blood glucose levels. After being diagnosed, Mirta’s concerns for her health have negatively affected her psychophysical well-being.

Mirta reports that she wakes up at dawn worrying whether her family feels well. These concerns lead her to make several phone calls to make sure that everybody is alright. Initially, these episodes were sporadic, but now they have become almost an obsession. Indeed, every morning, Mirta picks up the phone and starts calling her loved ones.

Mirta feels that doing these calls gives her more control over her worry; however, she realises that this worry has become a little invalidating, since she knows that without the morning phone call her anxiety would not disappear.”
