# Supplementary material for: Early Development of a Virtual Coach for Healthy Coping Interventions in Type 2 Diabetes Mellitus: Validation Study
Source: JMIR Form Res. 2022 Feb 11;6(2):e27500. doi: 10.2196/27500 (PMC8881774; doi:10.2196/27500)
Supplement: Multimedia Appendix 2 [file formative_v6i2e27500_app2.docx]

Multimedia Appendix 2

Table S1. Means and Standard Deviations of the positive items every fortnight week (second, fourth, sixth week) (N=18)

| Means (SD) | | | | | | |
| --- | --- | --- | --- | --- | --- | --- |
|  | Pleasant | Profound | Cordial | Comprehensible language | Empathetic | Attentive |
| Week 2 | 4.06 (.639) | 3.28 (.669) | 4.72 (.575) | 4.61 (.698) | 3.78 (.943) | 3.94 (.725) |
| Week 4 | 3.89 (1.079) | 3.00 (.907) | 4.78 (.548) | 4.67 (.485) | 3.56 (.705) | 4.00 (.767) |
| Week 6 | 4.11 (.900) | 3.28 (.752) | 4.78 (.428) | 4.78 (.428) | 3.67 (.907) | 3.72 (.575) |
|  | Motivating | Encouraging | Supportive | Trustworthy | Flexible | Interesting |
| Week 2 | 4.11 (.583) | 4.28 (.669) | 4.22 (.732) | 4.11 (.676) | 3.56 (.784) | 4.11 (.676) |
| Week 4 | 4.06 (.873) | 4.11 (.676) | 4.06 (.802) | 4.00 (.686) | 3.44 (.984) | 3.94 (.725) |
| Week 6 | 4.17 (.786) | 4.28 (.669) | 4.28 (.752) | 4.00 (.686) | 3.50 (.618) | 4.00 (.767) |

Table S2. Means and Standard Deviations of the negative items every fortnight week (second, fourth, sixth week) (N=18)

|  | Means (SD) | | | | | | |
| --- | --- | --- | --- | --- | --- | --- | --- |
|  | Annoying | Not reliable | Unappealing | Unclear | Complicated | Not efficient | Too much Information |
| Week 2 | 1.72 (.752) | 1.78 (1.003) | 1.94 (.802) | 1.39 (.608) | 1.44 (.616) | 1.61 (.850) | 1.89 (.676) |
| Week 4 | 1.89(1.079) | 1.39 (.608) | 2.11 (1.183) | 1.28 (.461) | 1.33 (.594) | 1.83 (.985) | 2.11 (.963) |
| Week 6 | 1.56 (.784) | 1.28 (.575) | 1.72 (.826) | 1.17 (.383) | 1.5 (.786) | 1.33 (.485) | 1.72 (.575) |
|  | Dissuading | Not stimulating | Not engaging | Unpredictable | Not reflective | Conventional | Rigid |
| Week 2 | 2.5 (1.043) | 1.94 (.802) | 2.00 (.907) | 2.33 (1.138) | 1.83 (.924) | 2.72 (.752) | 2.5 (.985) |
| Week 4 | 2.28 (.958) | 2.22 (1.003) | 2.00 (.907) | 1.72 (.669) | 2.39 (1.145) | 2.22 (.878) | 2.89 (.832) |
| Week 6 | 2.39 (.698) | 1.83 (.857) | 2.11 (.832) | 1.61 (.608) | 1.78 (.548) | 2.72 (.895) | 2.83 (.924) |
